# Complement Regulation in Immortalized Fibroblast-like Synoviocytes and Primary Human Endothelial Cells in Response to SARS-CoV-2 Nucleocapsid Protein and Pro-Inflammatory Cytokine TNFα

**DOI:** 10.3390/life12101527

**Published:** 2022-09-30

**Authors:** Vincent Franke, Sophie Meyer, Gundula Gesine Schulze-Tanzil, Tobias Braun, Maria Kokozidou, Theodor Fischlein, Sandeep Silawal

**Affiliations:** 1 Institute of Anatomy and Cell Biology, Paracelsus Medical University, Prof. Ernst Nathan Str. 1, 90419 Nuremberg, Germany; 2 Department of Cardiac Surgery, Cardiovascular Center, General Hospital Nuremberg and Paracelsus Medical University, Breslauer Str. 201, 90471 Nuremberg, Germany

**Keywords:** SARS-CoV-2, synovial fibroblasts, TNFα, nucleocapsid protein, complement factors, C5aR1, CD46, CD55, CD59, IL-6, endothelial cells, rheumatoid arthritis

## Abstract

*Background*: Case reports are available showing that patients develop symptoms of acute arthritis during or after recovery from SARS-CoV-2 infection. Since the interrelation is still unknown, our aim was to study the impact of the SARS-CoV-2 nucleocapsid protein (NP) on human fibroblast-like synoviocytes and human endothelial cells (hEC) in terms of complement and cytokine regulation. *Methods*: Non-arthritic (K4IM) synoviocyte, arthritic (HSE) synoviocyte cell lines and primary hEC were stimulated with recombinant NP and/or TNFα. Analyses of cell viability, proliferation, gene and protein expression of cytokines and complement factors were performed. *Results*: NP suppressed significantly the vitality of hEC and proliferation of HSE. NP alone did not induce any significant changes in the examined gene expressions. However, NP combined with TNFα induced significantly higher TNFα in HSE and K4IM as well as higher IL-6 and CD55 gene expression in HSE and suppressed C3aR1 gene expression in hEC. HSE proliferated twice as fast as K4IM, but showed significantly lesser gene expressions of CD46, CD55, CD59 and TNFα with significantly higher IL-6 gene expression. CD35 gene expression was undetectable in K4IM, HSE and hEC. *Conclusions:* NP might contribute in combination with other inflammatory factors to complement regulation in arthritis.

## 1. Introduction

The SARS-CoV-2 virus belongs to the Coronaviridae family of the Nidovirales order, which consists of enveloped, non-segmented, positive-sense, single-stranded RNA viruses [1]. Coronavirus particles contain four structural proteins: the spike (S), membrane (M), envelope (E), and nucleocapsid (N) proteins [1,2]. The most prominent structure is the club-shaped S protein on the surface of the virions. Within the double lipid membrane lies the nucleocapsid protein (NP), which has a helical, symmetrical structure [1,3,4]. In addition to its central role in the incorporation of genomic RNA into downstream virions, the NP also appears to interact strongly with the immune system. The specific immune response to the full-length N protein, as well as fragments of it, presented itself more pronounced than the immunoreactivity against fragments of the S or M proteins [5].

Severe cases of COVID-19 with high mortality are characterized by a massive production of pro-inflammatory cytokines, the so-called cytokine storm [6]. The elevated levels of circulating cytokines such as interleukin (IL)-6, tumor necrosis factor alpha (TNFα) or chemokines during the cytokine storm syndrome could promote hyperinflammation leading to acute respiratory disease or even multi-organ failure [7,8,9,10,11,12]. Even though COVID-19 is primarily manifested with respiratory symptoms, the involvement of extrapulmonary systems makes the disease highly unpredictable and variable. Extrapulmonary manifestations include those from the hematologic, gastrointestinal, renal, dermatologic, neurologic, and psychiatric systems [13]. Viral infections are also known to be capable of inducing autoimmune diseases, such as rheumatoid arthritis (RA) [14,15]. RA is a chronic, systemic autoimmune disease characterized by inflammatory arthritis, as well as extra-articular manifestations, usually affecting synovial joints. The disease is characterized by synovial membrane hyperplasia, hyperemia and inflammation. Hereby, the fibroblast-like synoviocytes adopt a typical invasive behavior associated with excessive proliferation, pannus formation on joint cartilage surface and cartilage erosions [16]. Over time, joint inflammation leads to destruction of the joint, cartilage, and bone [17,18]. Interestingly, several cases of anti-citrullinated peptide antibody (ACPA)-positive or ACPA-negative arthritic diseases have been reported after infection with SARS-CoV-2 virus, but the pathogenesis of these diseases is unknown to date [19,20,21]. Therefore, the discussion about occurrence of RA after COVID-19 remains controversial [19,22,23,24]. In addition, the course of COVID-19 in patients with pre-existing rheumatic diseases compared to healthy individuals in the general population is a matter of high concern. Grainger et al. addressed a moderately higher risk of infection in patients with inflammatory rheumatic disease in comparison to the general population [25]. The authors, however, also suggested that the small additional risk of poor outcomes could be mediated by any presence of comorbidities as well as immunomodulating medications in those patients [25]. Among many different protagonists in the immune response, the complement system is vigorously involved in attending immunological reactions against viral infections as well as autoimmune diseases [26,27].

The complement system is composed of humoral as well as membrane-attached proteins which are activated as a cascade via three different pathways depending on the context: indirectly via the classical pathway, directly via the lectin pathway, or spontaneously via the alternative pathway. All these pathways lead to a common terminal pathway to implement defense mechanisms, such as (I) opsonization of pathogens or infected cells by a proteolysis product of C3, C3b, (II) synthesis of anaphylatoxins such as C3a, C4a or C5a for inflammatory responses, or (III) the formation of membrane attack complexes (C5b-C9, MAC), also known as terminal complement complex (TCC), which form a pore in the cell membrane and lead to osmotic lysis of the cell [28,29,30]. Significant contribution of the complement system to the maintenance of immunological homeostasis can be seen with elimination of cellular debris as well as infectious pathogens via its rapid immune response. It is activated primarily by the recognition of “non-self” patterns and the ensuing cascade of enzyme reactions is tightly regulated by both soluble and membrane complement regulatory proteins (CRP) to protect against over-activation of the system. Inadequate regulation of the complement system leads to imbalance of the immunological homeostasis, resulting in either increased susceptibility to infectious, non-infectious or autoimmune diseases, such as RA [28,29,30,31].

Significantly elevated plasma concentrations of C3a, C3c, C5a and TCC were found in COVID-19 patients compared to healthy controls [32,33,34]. Strikingly, C3a and TCC were particularly elevated in patients with severe courses, such as respiratory failure, which can lead to death [33,35]. A preliminary report points out that SARS-CoV-2 NP is capable of activating the lectin pathway of the complement system via binding to mannose-binding protein-associated serine protease (MASP)-2 [36]. The S protein, in turn, directly activates the alternative pathway [37]. These activations may explain some of the clinical manifestations of COVID-19 that have been observed in other complement-driven diseases.

## 2. Materials and Methods

### 2.1. Cell Isolation, Cell Culture and Cell Stimulation 

Both SV40 T antigen immortalized synovial fibroblast cell lines, K4IM and HSE, were thankfully provided by Dr. Christian Kaps. These cells were cultivated using 10% fetal bovine serum (FBS) (PAN-Biotech, Aidenbach, Germany) supplemented growth medium. The growth medium consisted of Dulbecco’s MEM/Ham’s F-12 (1:1) with 25 mg/mL ascorbic acid, 50 IU/mL streptomycin, 50 IU/mL penicillin, 2.5 μg/mL amphotericin B and essential amino acids (all products: Carl Roth GmbH, Karlsruhe, Germany). A total of 0.05% trypsin/1.0 mM EDTA (Carl Roth GmbH) was used for passaging the cells before seeding them for the cell stimulation. After a 48-hour (h) adherence period, 1 h prior to the stimulation, the cells were starved with only 1% FBS supplemented respective growth medium. Same constitution was also used for diluting the stimulating reagents. Recombinant TNFα (Peprotech, Princeton, NJ, USA) and recombinant SARS-CoV-2 NP (R&D Systems, Minneapolis, MN, USA) were used as stimulating reagents. The cells were stimulated with these reagents ultimately for 24 h. 

To isolate primary hEC, remnants of the great saphenous vein from coronary bypass surgery were used. Sampling of human vein tissue was approved by both patient and the local ethics committee of the Bavarian Medical Association, Munich, Germany (ethical committee approval number: 16112). The vein samples were cannulated at both ends and equipped with three-way valves, allowing filling of the sample with 0.1% collagenase II (MP Biomedicals, Irvine, CA, USA) solution. The veins were incubated in 37 °C phosphate-buffered saline (PBS) for 30 minutes (min), then rinsed with 20 mL inactivation medium consisting of M199 (Sigma-Aldrich, St. Louis, MO, USA) and 20% FCS while the vessels were gently stimulated with the fingertips. The inactivation medium was centrifuged (5 min, 300 × *g*), decanted and the resulting pellet was resuspended in 5 mL of culture medium composed of inactivation medium and 36.4 ng/mL (2%) basic fibroblast growth factors (bFGF, Corning Incorporated, Corning, NY, USA). The obtained hEC were further cultivated in a growth medium (M199) with 20% FBS, 2% basic bFGF and heparin (Panpharma, Trittau, Germany) (50 U/mL each). Additionally, Gentamycin (Carl Roth GmbH) and Amphotericin B were added to the medium. hEC in passages four or five were used for the experiments.

### 2.2. Viability Assays

#### 2.2.1. Vitality Staining of Synovial Cells

K4IM and HSE fibroblasts were cultivated on Poly-L-Lysine (Biochrom AG, Berlin, Germany) coated coverslips. The stimulation was removed and washed with Hanks’ balanced salt solution (Thermo Fisher Scientific, Waltham, MA, USA) before a 50 µL solution of 0.5% fluorescein diacetate (FDA) (Sigma-Aldrich) and 0.1% propidiumiodide (Thermo Fisher Scientific) dissolved in PBS were pipetted onto the cells. After 3 min of incubation at room temperature (RT), the cells were assessed under confocal laser scanning microscope (Leica TCS SPEII and DMi8, Leica Microsystems, Wetzlar, Germany).

#### 2.2.2. CellTiter96^®^ Aqueous One Solution Cell Proliferation Assay

The CellTiter 96^®^ AQueous One Solution Cell Proliferation Assay (Promega Corporation, Walldorf, Germany) is used to determine cytotoxicity of the different stimulants in hEC. At a concentration of 10,000 cells/well (96-well plate), hEC were seeded in quintets and cultivated for 48 h. After the starving period of 1 h, the cells were stimulated with 1 µg/mL NP in starving medium for 24 h. The 10% DMSO (Carl Roth GmbH) in the starving medium served as a positive control. One hour before the end of stimulation, 20 µL of CellTiter 96^®^ AQueous One Solution reagent (3-(4,5-dimethylthiazol-2-yl)-5-(3-carboxymethoxyphenyl)-2-(4-sulfophenyl)-2H-tetrazolium, inner salt; MTS) was added to the cells in 100 µL of stimulation medium. After incubation at 37 °C for 1 h, absorbance of the samples was measured at 490 nm reference of 630 nm using a plate reader (Infinite M200 Pro, Tecan, Groedig, Austria).

#### 2.2.3. CyQUANT^®^ NF Cell Proliferation Assay

CyQUANT^®^ NF Cell Proliferation Assay (Thermo Fisher Scientific) was assessed to quantify the DNA concentration in the synovial fibroblasts under different conditions. Cell concentrations of 500 (HSE), 2000 as well as 7500 cells/well (HSE and K4IM) in quintets were seeded in a 96-well plate. A serial dilution of calf thymus DNA stock solution (1 mg/mL, Sigma-Aldrich) with TE-buffer (TRIS EDTA buffer, 10 mM TRIS [pH 8.0]; 1 mM EDTA in H_2_O_deionized_) was used as the standard curve. After stimulation completed, the cells were washed carefully with 1 × HBSS. Dye binding solution in 1 × HBSS was applied in the cell seeded wells, as well as the standard wells, as instructed by the manufacturer. The plates were incubated at 37 °C for 60 min. The fluorescence of each well was ultimately measured at 485_Ex_/530_Em_ nm in a plate reader (Infinite^®^ M200 Pro).

### 2.3. Gene Expression Analysis

#### 2.3.1. RNA Isolation and cDNA Synthesis

After the stimulation cells were rinsed with PBS. For lysing the cells, 1:100 solution of β-Mercaptoethanol (Sigma-Aldrich) in RNeasy Lysis Buffer (Qiagen, Hilden, Germany) was used. Total RNA was isolated using RNeasy Mini Kit (Qiagen) using the protocol provided by the manufacturer. Finally, RNA quantity was assessed with the NanoDrop^TM^ 2000 Spectralphotometer (Thermo Fisher Scientific). cDNA synthesis was performed with Mastercycler^®^ (Eppendorf, Hamburg, Germany) using the QuantiTect Transcription Kit (Qiagen) as instructed by the manufacturer.

#### 2.3.2. qPCR

Real time detection polymerase chain reaction (qPCR) analyses were performed using glyceraldehyde-3-phosphate dehydrogenase (GAPDH) as a reference gene and specific primers from TaqMan^TM^ Gene Expression Assays (Applied Biosystems, Foster City, CA, USA) listed in Table 1. cDNA template (20 ng cDNA/well, 1 µL) was mixed to TaqMan^TM^ Gene Expression Master Mix (Applied Biosystems) and water solution (9 µL) to perform the TaqMan^TM^ Gene Expression Assay. The TaqMan^TM^ analyses standard protocol was used and was carried out in Applied Biosystems StepOnePlus^TM^ Real-Time PCR System (Applied Biosystems). Relative gene expression levels were calculated with the 2-deltaCT method with normalization against the reference gene (51).

### 2.4. Protein Expression Analysis

For immunofluorescence labeling of the fibroblast-like synoviocytes 10,000 cells/well K4IM and 5000 cells/well (24-well plate, respectively) HSE fibroblasts were cultivated on Poly-L-Lysine coated coverslips. After the stimulation (Section 2.2), the cells were rinsed with Tris buffered saline (TBS, Medicago AB, Uppsala, Sweden) and fixated with 4% paraformaldehyde solution (PFA, Morphisto, Frankfurt, Germany) for 15 min. After rinsing, the cells were incubated in blocking buffer solution: 5% protease-free donkey serum (Chemicon, Temecula, CA, USA) diluted in TBS with 0.1% Triton X100 (Sigma-Aldrich) at RT for 20 min. A 50 µL solution with primary antibodies (Table 2) was applied to the cells and fixed cells were incubated in a humidifier chamber overnight at 4 °C, alternatively for 60 min at RT. The primary antibodies were omitted during the staining procedure for the negative controls. The cells were then rinsed thoroughly with TBS before secondary antibodies were applied and incubated again overnight at 4 °C or for 60 min at RT. Additionally, cell nuclei were counterstained using 4′,6-diamidino-2-phenylindole (DAPI) (Roche Diagnostics GmbH, Basel, Switzerland). Finally, after the final washing process with TBS the coverslips were mounted with Fluoromount G (SouthernBiotech, Birmingham, AL, USA) on the microscope slides. Images of the immunolabeled cells were taken using the confocal laser scanning microscopy. Finally, semiquantitative analysis of protein expression in stained images was performed by using ImageJ (US National Institutes of Health, Bethesda, MD, USA). Corrected total cell fluorescence (CTCF) values were calculated for the analysis measuring fluorescence intensity projected by cells [38]. The determination of Ki67 positive cells was performed manually using respective binary images.

### 2.5. Statistical Analysis

The statistical analyses were performed using GraphPad Prism (Version 8.1.4) (GraphPad Software, San Diego, CA, USA). The Shapiro–Wilk normality test was performed. Normalized data was expressed as the mean with standard deviation (mean ± SD). Differences between experimental groups were considered significant at *p* < 0.05 as determined by one sampled t-test. ANOVA analyses were performed using the Tukey’s multiple comparisons test. The ROUT test was applied to identify the outliers which were excluded from the statistics.

## 3. Results

### 3.1. Vitality Assays

**Figure 1:**
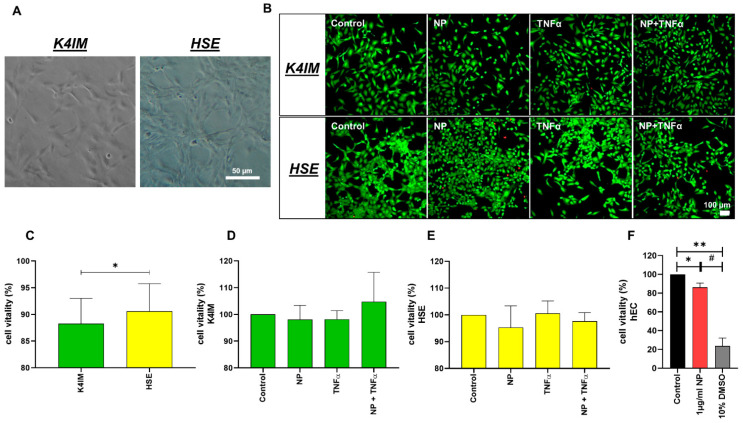
(**A**) Native images of synoviocyte cell lines, K4IM and HSE (100 × magnification, scale bar = 100 µm). (**B**) Representative overlay images of live-dead staining of the synoviocytes after 24 h stimulation with 1 µg/mL NP, 10 ng/mL TNFα or the combination. Fluorescein diacetate (green: vital cells) and propidium iodide (red: dead cells) have been used for staining. (**C**) Comparison of the vitality of unstimulated K4IM and HSE after 24 h, analyzed using ImageJ. n = 4. (**D**,**E**) Comparison of the vitality of stimulated K4IM and HSE, respectively, after 24 h stimulation. Number of living cells in relation to total cells (100%) measured in live-dead staining images of the synoviocytes, with control normalized to 100 as reference. D (n = 3), E (n = 4). (**F**) Cytotoxicity was assessed (CellTiter 96® AQueous One Solution Cell Proliferation Assay) on the hEC after 24 h stimulation with 1 µg/mL NP and 10% DMSO as positive control. n = 3. Control has been normalized to 100. Mean and the standard deviation (SD) are depicted. One sample *t*-test with significance in relation to control (*). Repeated Measures one-way ANOVA using Tukey’s multiple comparisons (#). */# = *p* ≤ 0.05; ** = *p* ≤ 0.01.

Native histological images showed that the HSE cell line proliferated vigorously showing close cell–cell contact in comparison to K4IM fibroblasts (Figure 1A). Unstimulated HSE synovial fibroblasts displayed a slightly higher cell vitality compared to unstimulated K4IM fibroblasts using the vitality staining (Figure 1B,C). The 1 µg/mL NP as well as the 10 ng/mL TNFα stimulations of K4IM as well as HSE did not induce any significant changes analyzed in the live-dead images (Figure 1B,D,E). CellTiter 96^®^ AQueous One Solution Cell Proliferation Assay was used to analyze the viability in response to stimulation with 1 µg/mL NP in comparison to the control group in hEC (Figure 1F). Although significant, a less than 15% decrease in vitality, however, was observed under stimulation with 1 µg/mL NP in comparison to the non-stimulated cells. A positive control stimulation with 10% DMSO displayed a high toxicity with less than 25% cell vitality (Figure 1F).

### 3.2. DNA Quantification and Cell Proliferation

**Figure 2:**
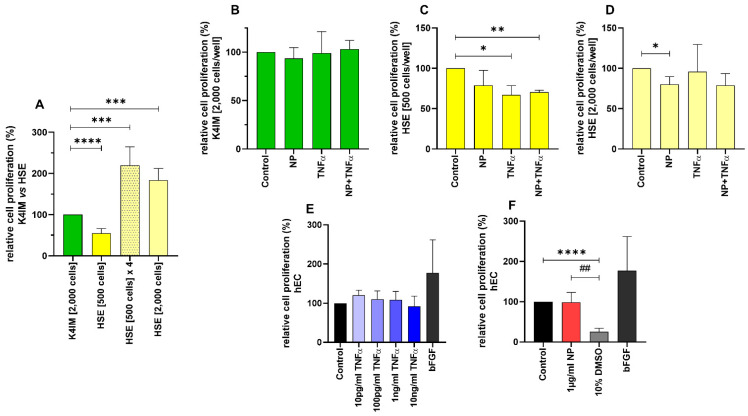
Comparison of the relative cell proliferation in (**A**) unstimulated (n = 8), (**B**–**D**) stimulated K4IM and HSE cell lines (n = 4, 1 µg/mL NP, 10 ng/mL TNFα or the combination) as well as (**E**,**F**) stimulated human endothelial cells (hEC) assessed by CyQUANT® NF Cell Proliferation Assay. E (n = 4), F (n = 5). Mean with standard deviation (SD). Control has been normalized to 100. One sample *t*-test with significance in relation to control (*). Repeated Measures one-way ANOVA using Tukey’s multiple comparisons (#). * = *p* ≤ 0.05; **/## = *p* ≤ 0.01; *** = *p* ≤ 0.001, **** = *p* ≤ 0.0001.

CyQUANT^®^ NF Cell Proliferation Assay showed almost double DNA content in non-stimulated HSE fibroblasts in comparison to non-stimulated K4IM after 3 days of cultivation when 2000 cells/well were seeded (Figure 2A). A less dense seeding with 500 cells/well HSE was undertaken, and the DNA content was about half compared to the K4IM cell line, where 2000 cells/well were seeded in. After 3 days of cultivation, it could be seen that the DNA content had already been heightened to half the amount of the K4IM group. By implication, with mathematical compensation of the seeded cell count, once again the theoretical DNA content of HSE was about double compared to K4IM (Figure 2A: HSE [500 cells] × 4). 

In the K4IM cell line, no significant differences could be detected after the stimulation (Figure 2B). Interestingly, different results could be gained in the HSE cell line depending on the density of the seeded cells. In the HSE group with 500 cells/well seeding, all the stimulations showed a reduced DNA content in comparison to the control group, hereby TNFα with and without NP showed statistically significant results (Figure 2C). In the HSE group with 2000 cells/well, a significant decrease with NP stimulation was detected compared to the control (Figure 2D). No significant result in this densely seeded HSE group was detected when the cells were stimulated with TNFα (with or without NP) in comparison to the less dense HSE group.

CyQUANT^®^ NF Cell Proliferation Assay of hEC showed no significant cell proliferative effect when these cells were stimulated with NP. The positive control with 10% DMSO displayed a drastic decrease in the DNA content, which proves the validity of this assay, since the cytotoxic effect of DMSO is already known. Likewise, the stimulation with bFGF demonstrated an increased cell proliferation, however it did not reach the significance level in hEC (Figure 2F).

**Figure 3:**
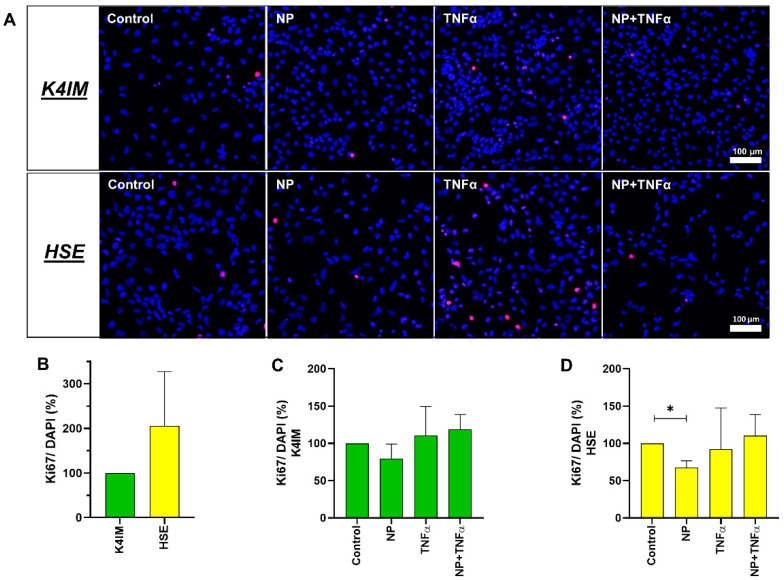
(**A**) Representative overlay of immunofluorescence images of Ki67-stained unstimulated and stimulated K4IM and HSE fibroblasts (200× magnification, 1 µg/mL NP, 10 ng/mL TNFα or the combination). Red (Cy3) = Ki67, blue (DAPI) = cell nuclei. n = 3. (**B**–**D**) Graphic representation of the ratio between proliferation marker Ki67-positive cells and total cells (DAPI) in percentage, mean with standard deviation (SD). One sample t-test with significance in relation to control (*). Repeated Measures one-way ANOVA using Tukey’s multiple comparisons. * = *p* ≤ 0.05. Scale bar = 100 µm.

Cell proliferation was also analyzed using the images of cells immunohistochemically stained with anti-Ki67 antibody and DAPI. The analysis indicated a trend of an increased cell proliferation rate of the HSE cell line compared to the K4IM cell line (Figure 3A,B) (non-significant). Stimulation of HSE with NP displayed a significant decrease in cell proliferation (Figure 3D). A similar effect, however non-significant, could be seen in K4IM (Figure 3C).

### 3.3. Gene Expression

**Figure 4:**
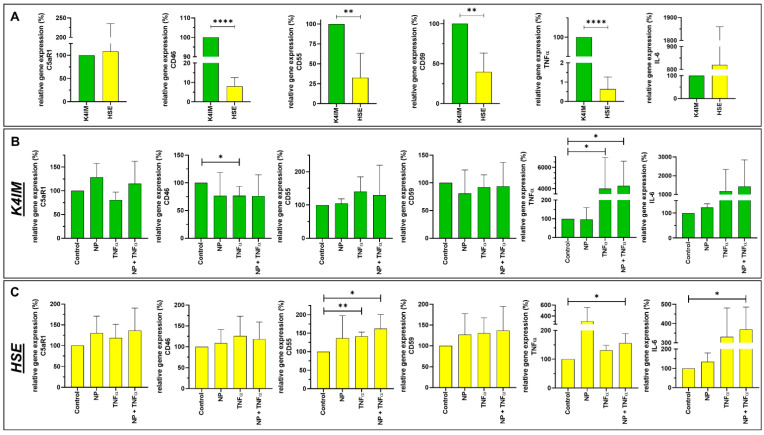
Graphic representation of relative gene expression of complement factors (C5aR1, CD46, CD55 and CD59) and cytokines (TNFα and IL-6) in non-stimulated synoviocyte cell lines K4IM and HSE (**A**) as well as K4IM (**B**) or HSE (**C**) when stimulated separately for 24 h with 1 µg/mL NP, 10 ng/mL TNFα or the combination of both. n = 3–5. Mean with standard deviation (SD). Control has been normalized to 100. One sample *t*-test with significance in relation to control (*). Repeated Measures one-way ANOVA using Tukey’s multiple comparisons. * = *p* ≤ 0.05, ** = *p* ≤ 0.01, **** = *p* ≤ 0.001.

K4IM and HSE showed a non-significant difference in C5aR1 gene expression. In the case of the complement regulatory proteins, CD46, CD55 and CD59 gene expression was lower in HSE in comparison to K4IM. CD35 gene expression could not be detected in K4IM, HSE and hEC (not shown). The highest difference in gene expression was found in CD46, where the gene expression in HSE was detected as being 10-fold lower than in K4IM (Figure 4A). Furthermore, K4IM showed circa 4-fold and 2-fold higher CD55 and CD59 gene expression, respectively, in comparison to HSE. More than 100-fold lower TNFα gene expression was observed in HSE in comparison to K4IM, whereas HSE displayed circa 8-fold higher gene expression of IL-6 in comparison to K4IM (non-significant) (Figure 4A).

NP alone did not induce any significant changes in examined complement or cytokine gene expression in fibroblast-like synoviocytes or hEC (Figure 4B,C, Supplementary Figure S1). TNFα alone, however, suppressed CD46 gene expression in K4IM, but not in HSE (Figure 4B,C). Similarly, TNFα alone or in combination with NP induced more than 400-fold TNFα gene expression in K4IM, whereas there was only about 1.5 fold increase in gene expression in HSE via stimulation of TNFα in combination with NP. Combined stimulation with TNFα and NP also showed drastically higher IL-6 gene expression in HSE. A similar trend could be determined in K4IM cells where TNFα alone or in combination with NP induced more than 10-fold elevated IL-6 gene expression compared to the control group (non-significant). In hEC, the combined stimulation led to a high suppression in C3aR1 gene expression in comparison to the control group (Supplementary Figure S1).

### 3.4. Protein Synthesis

**Figure 5:**
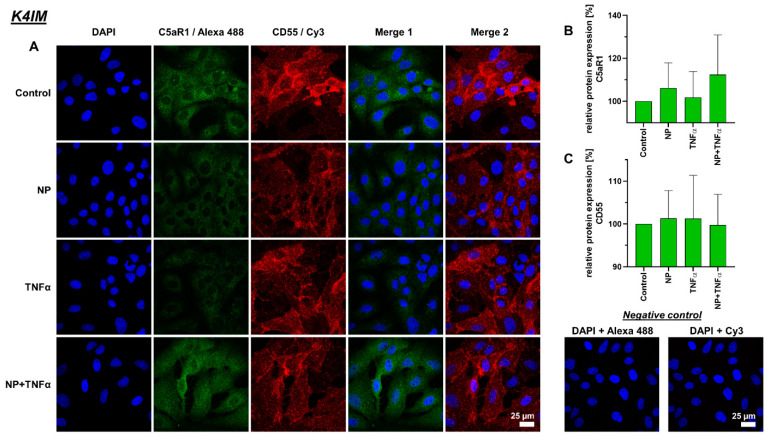
(**A**) Representative images of K4IM cell line after 24 h stimulation (630× magnification, 1 µg/mL NP, 10 ng/mL TNFα or the combination), immunolabeled with C5aR1 and CD55 specific antibodies and negative control of the staining. Green (Alexa 488) = C5aR1, red (Cy3) = CD55, blue (DAPI) = cell nuclei. Scale bar = 25 µm. Graphic representation of relative (**B**) C5aR1 and (**C**) CD55 protein fluorescence intensity, n = 4, mean with standard deviation (SD). Control has been normalized to 100. One sample t-test with significance in relation to control. Repeated Measures one-way ANOVA using Tukey’s multiple comparisons.

**Figure 6:**
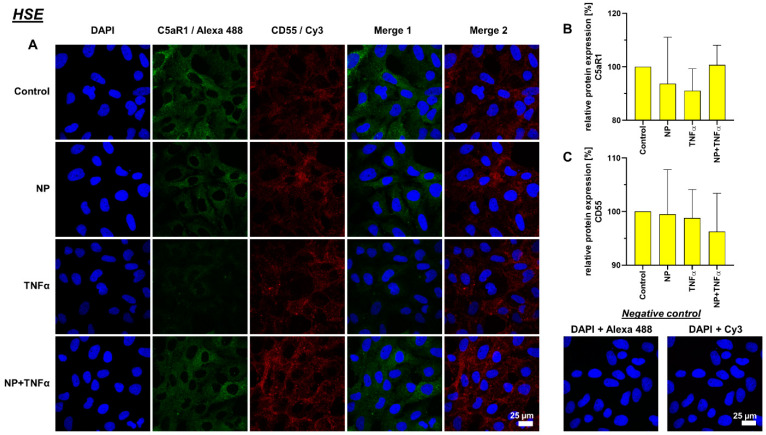
(**A**) Representative images of HSE cell line after 24 h stimulation (630× magnification, 1 µg/mL NP, 10 ng/mL TNFα or the combination), immunolabeled with C5aR1 and CD55 specific antibodies and negative control of the staining. Green (Alexa 488) = C5aR1, red (Cy3) = CD55, blue (DAPI) = cell nuclei. Scale bar = 25 µm. Graphic representation of relative (**B**) C5aR1 and (**C**) CD55 protein fluorescence intensity, n = 4, mean with standard deviation (SD). Control has been normalized to 100. One sample t-test with significance in relation to control. Repeated Measures one-way ANOVA using Tukey’s multiple comparisons.

**Figure 7:**
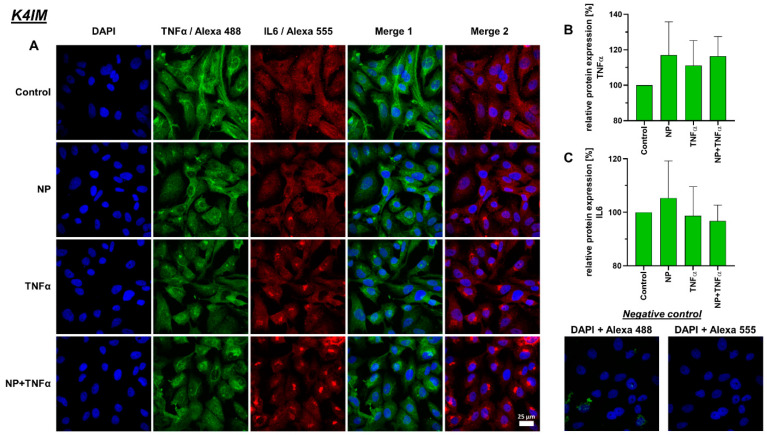
(**A**) Representative images of K4IM cell line after 24 h stimulation (630× magnification, 1 µg/mL NP, 10 ng/mL TNFα or the combination), immunolabeled with TNFα and IL-6 specific antibodies and negative control of the staining. Green (Alexa 488) = TNFα, red (Alexa 555) = IL-6, blue (DAPI) = cell nuclei. Scale bar = 25 µm. Graphic representation of relative (**B**) TNFα and (**C**) IL-6 protein fluorescence intensity, n = 3, mean with standard deviation (SD). Control has been normalized to 100. One sample t-test with significance in relation to control. Repeated Measures one-way ANOVA using Tukey’s multiple comparisons.

**Figure 8:**
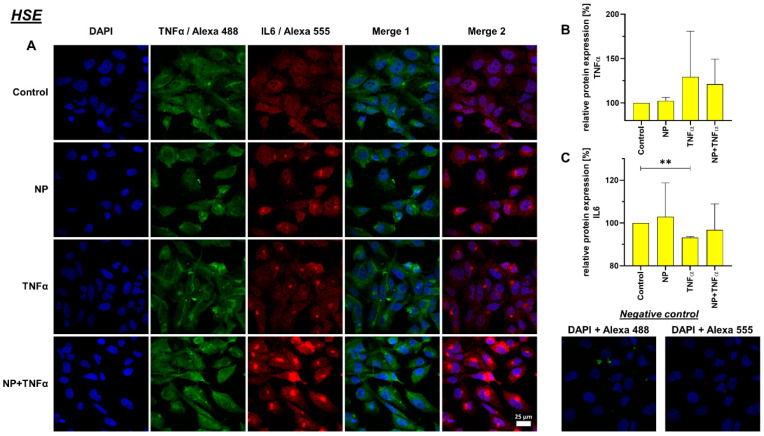
(**A**) Representative images of HSE cell line after 24 h stimulation (630× magnification, 1 µg/mL NP, 10 ng/mL TNFα or the combination), immunolabeled with TNFα and IL-6 specific antibodies and negative control of the staining. Green (Alexa 488) = TNFα, red (Alexa 555) = IL-6, blue (DAPI) = cell nuclei. Scale bar = 25 µm. Graphic representation of relative (**B**) TNFα and (**C**) IL-6 protein fluorescence intensity, n = 3, mean with standard deviation (SD). Control has been normalized to 100. One sample *t*-test with significance in relation to control (**). Repeated Measures one-way ANOVA using Tukey’s multiple comparisons. ** = *p* ≤ 0.01.

Intra- and extracellular protein expression of C5aR1, CD55, TNFα and IL-6 were evaluated using immunofluorescence staining (Figure 5, Figure 6, Figure 7 and Figure 8). C5aR1 showed a uniform distribution of protein in both cell lines, however, the expression was weak in comparison to other examined proteins (Figure 5 and Figure 6). In comparison, CD55 expression was dominantly present in both cell lines. However, no stimulation-related significant results were obtained (Figure 5 and Figure 6).

A decreased IL-6 protein synthesis under TNFα and NP + TNFα stimulation compared to the control was depicted, where the stimulation of HSE fibroblasts with only TNFα even showed significant results. In staining, K4IM as well as HSE cell lines displayed perinuclear accumulation of this protein (Figure 7 and Figure 8).

## 4. Discussion

Commonly, fibroblast-like synoviocytes in RA proliferate in an anchorage-independent manner and have furthermore, impaired contact inhibition [39,40]. Additionally, these cells are more robust as a result of low rate of apoptosis supported by a higher expression of pro-survival factors [41]. This is in agreement with the significantly higher vitality in the HSE cell line in comparison to K4IM detected in the present study. We observed a significant difference in the proliferative activity of these two synoviocyte cell lines. HSE built compact clusters in comparison to K4IM, explaining the low contact inhibition in these cells as it is shown in the native images (Figure 1A). As mentioned above, the inhibition of apoptosis could be partially responsible for the synovial hyperplasia observed in RA [41]. Even though NP stimulation reduced the cell vitality in K4IM and HSE slightly, these results were not significant. In case of hEC, CellTiter 96^®^ AQueous One Solution Cell Proliferation Assay showed a minimal but significant increase in cytotoxicity when the cells were stimulated with 1 µg/mL NP. Yet still, the overall cell vitality remained over 85% compared to the control group. Hence, the histopathological observations of severe endothelial damage with ruptured cell membranes, and perivascular T-cell infiltration in SARS-CoV-2 infections cannot be fully related to NP [8]. As expected, the positive control with 10% DMSO stimulation demonstrated toxic effect on the hEC compared to non-stimulated group as well as 1 µg/mL NP stimulation group. This effect was described in a 2017 publication outlining the increased apoptosis rate of human umbilical vascular endothelial cells line EAhy926 cells after stimulation with 0.2%, 0.4% and 0.6% DMSO [42].

Because of the observed vigorous cell proliferation in HSE, CyQUANT^®^ NF Cell Proliferation Assay was performed using two different seeding densities of HSE fibroblasts for this assay. K4IM fibroblasts were seeded at a concentration of 2000 cells/well; whereas, in HSE, two different densities, namely 500 cells/well and 2000 cells/well, were used. After 3 days of cultivation, it was shown that the HSE DNA content was double the amount compared to K4IM, explaining the higher proliferation activity of these cells. The various stimulations of K4IM did not show significant changes in the proliferation pattern. In comparison, NP alone could significantly reduce the proliferation in HSE when 2000 cells/well were seeded, whereas in lower cell density, the effect was only found as a trend. Since in arthritis synovial fibroblasts form a multilayer, this inhibitory effect might be of relevance in vivo but could be interpreted to be protective in RA. TNFα with and without NP reduced the cell proliferation in HSE when the cells were seeded in a 500 cells/well density. In case of hEC, NP did not induce any significant changes related to cell proliferation. This could be interpreted as that the transformed arthritic cells are perhaps susceptible to the influence of NP in terms of cell proliferation in comparison to the non-arthritic cells. There are reports available where it is mentioned that isolated rheumatic synovial fibroblasts show increased tumor-like growth [43,44]. Immunofluorescence staining with Ki67 showed a trend of higher proliferative activity of HSE in comparison to K4IM, but significantly confirmed the suppressive effect of NP. Since Ki67 is expressed in the nuclei of proliferating cells, Pessler et al. analyzed the enhanced subintimal Ki67 expression in inflammatory arthropathies compared to normal controls and correlated it positively with the histopathological severity of synovitis [45]. This behavior of rheumatic synovial fibroblasts, together with the observations of viability, can be related to synovial hyperplasia and pannus formation in RA [46]. The overall consequence of impaired fibroblast proliferation by NP shown in the HSE cell line could rather suggest a protective effect of NP in RA. A strong and clearly significant decrease in proliferation was only detected when cells were stimulated with 10% DMSO, which is again consistent with previously described toxicity of DMSO, which blocks the cell cycle at the transition from G1 to S phase [42]. 

During the process of complement cascade activation, anaphylatoxins C3a and C5a are produced as fragments of the complement proteins C3 and C5, respectively. These anaphylatoxins are pro-inflammatory polypeptides that exert their biologic activities through ligation of their respective receptors, i.e., C3aR1, C5aR1 and C5L2. In our study, NP induced a higher C5aR1 gene expression in the synoviocytes (not significant) and suppressed the C5aR1 gene expression in the hEC. NP + TNFα significantly lowered the C3aR1 gene expression in the hEC. In another study stimulation of glomerular and brain microvascular endothelial cells with 10 ng/mL TNFα for 48 h has shown a rather higher C3aR1 expression [47].

Complement receptor type 1, CD35, is a type I membrane protein expressed primarily in blood cells, glomerular podocytes, and follicular dendritic cells [48]. CD35 has multiple functions where it acts as a receptor for C3b and C4b. In addition, this receptor performs as a cofactor for Factor I, mediating the cleavage of C3b and C4b. Finally, it also helps to accelerate convertase decay regulating the complement activation [49]. Earlier immunohistochemical staining report of synovium from 1991 showed CD35 protein signals in subintimal macrophages, but not in synovial lining cells [50]. Consistent to this publication, CD35 gene expression could not be detected in fibroblast-like synoviocytes in our study. Furthermore, in hEC, CD35 gene expression was undetectable. This report is novel, since gene as well as protein expression of CD35 has been reported previously in human umbilical vein endothelial cells in passages three or lower, which showed to be increased under hypoxia [51]. Nevertheless, in the present study great saphenous vein-derived hEC from adult donors were investigated which might slightly differ in their expression profile.

In general, the gene expressions of other membrane attached CRP, i.e., CD46, CD55, and CD59 were significantly decreased in HSE compared to K4IM. The membrane cofactor protein (MCP), CD46, can both promote and inhibit inflammatory responses [52]. It is involved in the cleavage of C3b and C4b with proteolytic action and further serves self-defense of cells from the complement system. Another function is the downregulation of adaptive T-helper cells. Defective regulation of effector T cells represents a central issue in RA, establishing a link between CD46 dysregulation and RA [53]. CD46 gene expression in HSE was only about 10% the expression level of K4IM derived from healthy synovial fibroblasts. The reduced CD46 gene expression in HSE upon TNFα stimulation could suggest that inflammatory responses induce activation of the complement system by reducing these CRP. 

Disruptions in complement decay accelerating factor (DAF, CD55) expression impact a number of diseases, including RA [54]. CD55 inhibits complement activation by accelerating degradation of C3 convertase as well as inhibiting C3 convertase formation. CD55 that is deposited on the local collagen fiber meshwork in synovia tissue protects from immune complex-mediated arthritis [55]. In addition, CD55 serves as a ligand for CD97, which is expressed on monocytes and other blood-derived cells stimulating the proliferation of immune cells. This interaction has been suggested in the pathogenesis of RA [56,57]. In our study, we found a lowered CD55 gene expression in HSE compared to K4IM, where we could also suggest a reduced CD55 related protection of the cells against arthritis. However, on the other hand, a reduced CD55-CD97 interaction has also been implied to ameliorate the RA severity [58]. Hence, a clear role of CD55 in the pathogenesis of RA is still not clear. An increase in CD55 was detected after TNFα stimulation of intestinal epithelium, smooth muscle cells and endothelial cells [59,60,61]. Complementarily, we observed the same significant effect of increased CD55 gene expression in HSE when stimulated with TNFα with and without NP.

CD59 (protectin) is a regulatory protein that exerts inhibitory influence on the formation of the MAC [30]. The immunohistological study of RA synovium showed non-detectable CD59 protein expression in the synovial lining cells and relatively weak CD59 expression in stromal and endothelial cells [62]. In fact, our result showed that the HSE cell line has a reduced CD59 gene expression in comparison to K4IM. However, NP or TNFα stimulation did not induce any significant changes in the studied cells. In a rat model with collagen-induced arthritis, it was found that inhibition of CD59 further enhanced the inflammatory response, whereas intra-articular injection of CD59 reversed this effect [63]. 

TNFα is an important pro-inflammatory cytokine involved in acute and chronic inflammation, autoimmune diseases and tumors [64]. The synovium contains lining membrane and sublining tissue underneath. Fibroblasts such as synoviocytes with the synovial macrophages make the inner lining; whereas, the subintimal layer contains connective tissue with fibroblasts, macrophages, as well as vessels and nerves [65]. TNFα-containing cells tend to be localized in deeper, perivascular, interstitial areas of the synovium, rather than on the immediate surface of the synovial membrane [66]. About 70–90% of TNFα-producing cells in synovium express monocyte/macrophage markers as well, whereas only 0–2.5% express T-cell co-receptors [66]. The same study showed that no TNFα expression was detected in intimal synovial fibroblasts. In contrast to the measurement of increased TNFα levels in peripheral blood samples from RA patients, we observed that HSE have a significantly lower TNFα gene expression (about 1%) in comparison to that of K4IM. We could confirm that synovial fibroblasts stimulation with TNFα leads to an increase in inflammatory cytokines, i.e., approximately 40-fold increase in TNFα gene expression. Similar results have already been published before when K4IM fibroblasts were treated with 10 ng/mL TNFα either for 24 h under serum-starved conditions or for 30 min, washed, and further incubated in serum-starved medium for another 6 h [67,68]. The latter approach resulted in a peak relative TNFα expression of 5.0 ± 0.9 and an increase in IL-6 expression of 3.4 ± 0.4 after 6 h, respectively. A 40-fold increase in TNFα or non-significant 10-fold elevation of IL-6 gene expression in our study, could be a result of a continuous TNFα stimulation for 24 h. 

IL-6 is produced in response to infection as well as in tissue damage. It provides protection by stimulating acute phase, hematopoietic and immunologic responses. IL-6 expression is tightly regulated, therefore, it can contribute to chronic inflammation or autoimmunity, such as RA, when dysregulated [69,70]. IL-6 is highly elevated in RA, in both synovial fluid and isolated synovial tissue [71]. Fibroblast-like synoviocytes are considered as significant producers of IL-6, with even enhanced expression under stimulation of TNFα, IL-1β, prostaglandin E2, etc. [68,72]. In our study, we showed that gene expression of IL-6 in arthritic HSE is about 8-fold higher than non-arthritic K4IM. TNFα stimulation with or without NP induced an almost 10-fold increase in IL-6 in K4IM in comparison to the HSE with around 8-fold higher expression under its influence. The induction of IL-6 by TNFα has already been shown in a previous study and this effect was also detectable to a much higher degree in primary human synovial fibroblasts [68]. TNFα and NP + TNFα stimulated cells, K4IM as well as HSE fibroblasts, displayed increased numbers of perinuclear vesicles with intense IL-6 protein signal. The elevated IL-6 gene expression when stimulated with TNFα and NP + TNFα can be related to increased protein expression. IL-6 is constitutively expressed in RA and this overproduction may be partly responsible for both localized and generalized symptoms of the disease [71]. Kunisch et al. performed ELISA measurement using cell supernatants to detect 55-fold increased IL-6 release in RA, as well as an 82-fold increase in osteoarthritic synovial fibroblasts after stimulation with 10 ng/mL TNFα [73]. Therefore, not only TNFα inhibitors but also IL-6 inhibitors have been used for the therapy of RA since 2008 [74]. In cases of new-onset arthritis or worsening of pre-existing joint disease associated with COVID-19, IL-6 receptor inhibition was found to suppress inflammatory processes in joints [75].

Despite promising results, we still emphasize that the comparison in the complement and cytokine gene and protein expression between arthritic and non-arthritic cells should be performed in primary non-immortalized cells, since the immortalization process could still transform the original genotype and phenotype of the cells. A previous study comparing the cytokine response between the K4IM cell line and primary human synovial fibroblasts revealed a similar regulation suggesting K4IM to be a valid synovial fibroblast model, but it indicated also a more sensitive response for primary synovial fibroblasts [68]. Our study showed that NP alone could impair the cell proliferative activity in synoviocytes; however, no significant alteration of the selected complement as well as cytokine gene and protein expression could be detected, in contrast to the stimulation of NP combined with TNFα or TNFα alone. In the future, a direct co-culture approach including immune cells, such as macrophages or stimulation with other complement factors, has to be performed that achieve a more elaborate immunological overview in the joint. Beyond that, we believe that further research using different concentrations of the stimulants NP and TNFα should be performed, since we focused on rather high concentrations to determine whether there is any relevant effect at all. This concentration was based on a report [37] from saliva and serum, but the concentration in the synovial fluid and joint is unknown.

Furthermore, other viral proteins or RNA viral components of SARS-CoV-2 should be investigated to better understand the possible impact of the virus on the joint homeostasis.

## 5. Conclusions

The hypothesis that NP protein could impair cell viability was only proven for hEC. NP significantly impaired the cell proliferation in HSE fibroblasts, but this effect did not reach the significance level in K4IM.

In regard to cytokine and complement, NP alone did not induce any significant changes in the gene expression of the selected cytokines and complement components in fibroblast-like synoviocytes or hEC. However, NP combined with TNFα induced significantly higher TNFα in K4IM and HSE and IL-6 gene expression in HSE fibroblasts, and also significantly higher CD55 gene expression in HSE compared to the control. In hEC, the combined stimulation with NP and TNFα led to a suppression in C3aR1 gene expression in comparison to the control group. TNFα alone induced CD55 gene expression in HSE as well as TNFα gene expression in K4IM, but suppressed CD46 gene expression in K4IM. This suggests that NP might exert effects under inflammatory conditions.

In addition, distinct differences in the response of both synovial fibroblast lineages could be demonstrated: The immortalized arthritic synoviocytes, HSE, proliferate double the rate in comparison to K4IM, the immortalized non-arthritic synoviocytes. In general, a lower gene expression of complement factors such as CD46, CD55, CD59 and TNFα, but a higher IL-6 gene expression, was found in HSE in comparison to K4IM. CD35 gene expression could be detected neither in fibroblast-like synoviocytes nor in hEC.

Eventually, it seems unlikely that a high concentration of NP in the serum of COVID-19 patients alone induces synovial inflammation. The fact that NP reduces cell proliferation in HSE synoviocytes could be interpreted as a note on a protective effect of NP on synovioytes of RA patients, but it is more likely that increased risk of infection or poor outcome are related to the immune status and immunomodulating medication of patients with autoimmune diseases, such as RA.

## Figures and Tables

**Table 1:** Oligonucleotides used for qPCR analysis.

Primer	Company	Sequence	Assay ID	Amplicon Length (bp)
GAPDH	ABI	*	Hs99999905_m1	93
CD46	ABI	*	Hs00387246_m1	94
CD55	ABI	*	Hs00167090_m1	62
CD59	ABI	*	Hs00174141_m1	70
C3aR1	ABI	*	Hs00269693_s1	82
C5aR1	ABI	*	Hs00704891_s1	68
TNFα	ABI	*	Hs00174128_m1	80
IL-6	ABI	*	Hs00174131_m1	95

*: Sequence not provided by the company, ABI (Applied Biosystems, Foster City, CA, USA).

**Table 2:** Antibodies and dyes used.

Specificity and Species	Company	Catalogue Number	Stock Concentration	Used Dilution
Mouse anti-human C5aR1	GeneTex, Eching, Germany	GTX74845	1 mg/mL	1:50
Goat anti-human CD55	R&D systems, Minneapolis, MN, USA	AF2009	200 µg/mL	1:50
Rabbit anti-human IL-6	Novus Biologicals, Centennial, CO, USA	NB600-1131	^+^	1:50
Goat anti-human TNFα	Peprotech, Princeton, NJ, USA	500-P31AG	0.5 mg/mL	1:50
Mouse anti-human Ki67	Chemicon International Inc., Temecula, CA, USA	MAB4190	1 mg/mL	1:50
Donkey anti-mouse- Alexa Fluor 488	Invitrogen, Waltham, MA, USA	A21202	2 mg/mL	1:200
Donkey anti-goat- Alexa Fluor 488	Life Technologies, Carlsbad, CA, USA	A11055	2 mg/mL	1:200
Donkey anti-rabbit- Alexa Fluor 555	Life Technologies, Carlsbad, CA, USA	A31572	2 mg/mL	1:200
Donkey anti-goat- cyanine (Cy)3	Dianova, Hamburg, Germany	705-165-147	1.5 mg/mL	1:200
Donkey anti-mouse-Cy3	Dianova, Hamburg, Germany	715-166-150	1.5 mg/mL	1:200

^+^ not available.

## Data Availability

The data presented in this study are available in this article and its Supplementary Materials.

## Supplementary Materials

The following supporting information can be downloaded at: https://www.mdpi.com/article/10.3390/life12101527/s1, Figure S1: Graphic representation of relative gene expression of complement factors C3aR1 (A) and C5aR1 (B) in hEC after 24 h stimulation. n = 3. Mean with standard deviation (SD). Control has been normalized to 100. One sample t-test with significance in relation to control (*). Repeated Measures one-way ANOVA using Tukey’s multiple comparisons. * = *p* ≤ 0.05.

## Abbreviations

ACPA	Anti-citrullinated peptide antibody
bFGF	Basic fibroblast growth factor
C3aR1	C3a receptor 1
C5aR1	C5a receptor 1
cDNA	Complementary DNA
CRP	Complement regulatory protein
CTCF	Corrected total cell fluorescence
DAPI	4′,6-Diamidin-2-phenylindol
DMSO	Dimethyl sulfoxide
FBS	Fetal bovine serum
FDA	Fluorescein diacetate
GAPDH	Glyceraldehyde-3-phosphate dehydrogenase
h	Hour
HBSS	Hanks’ balanced salt solution
hEC	Human endothelial cells
HSE	Immortalized human rheumatoid arthritic fibroblast-like synoviocytes
IL	Interleukin
K4IM	Immortalized human non-arthritic fibroblast-like synoviocytes
MAC	Membrane attack complex
MAPK	Mitogen activated protein kinase
MASP-2	Mannose-binding protein-associated serine protease 2
Min	Minutes
NF-κB	Nuclear factor ‘kappa-light-chain-enhancer’ of activated B-cells
NP	Nucleocapsid protein
PAMP	Pathogen-associated molecular patterns
PBS	Phosphate-buffered saline
PFA	Paraformaldehyde solution
qPCR	Real time detection polymerase chain reaction
RT	Room temperature
SARS-CoV-2	Severe acute respiratory syndrome coronavirus type 2
TBS	Tris buffered saline
TCC	Terminal complement complex
TLR	Toll-like receptors
TNFα	Tumor necrosis factor
VEGF	Vascular endothelial growth factor
