# Supplementary figures and images for: Complement Regulation in Immortalized Fibroblast-like Synoviocytes and Primary Human Endothelial Cells in Response to SARS-CoV-2 Nucleocapsid Protein and Pro-Inflammatory Cytokine TNFα
Source: Life (Basel). 2022 Sep 30;12(10):1527. doi: 10.3390/life12101527 (PMC9604721; doi:10.3390/life12101527)

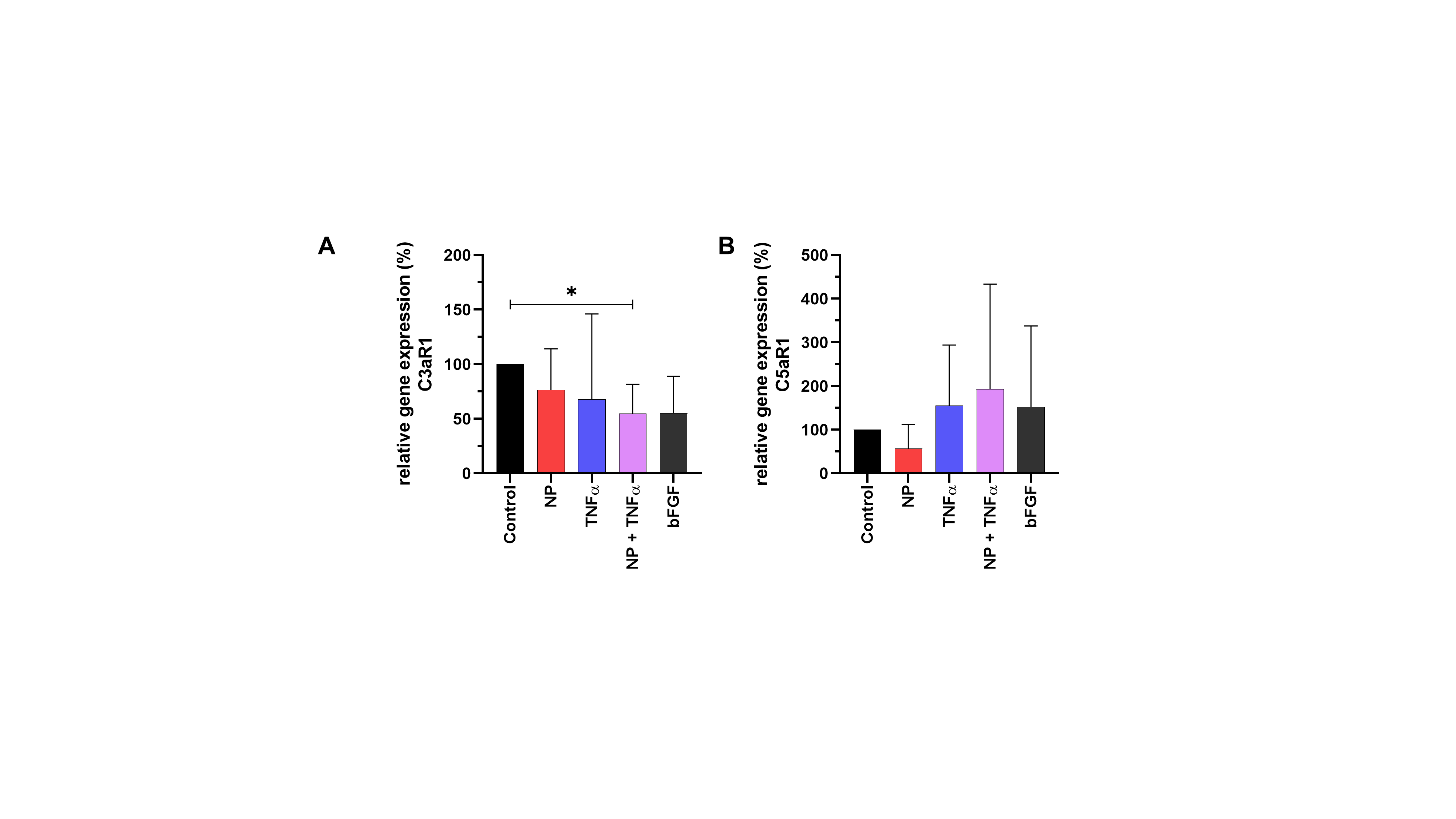

Supplement: Supplementary file 1 [file life-12-01527-s001.zip › life-1889470-supplementary.TIF]
